# Optogenetic Release of ACh Induces Rhythmic Bursts of Perisomatic IPSCs in Hippocampus

**DOI:** 10.1371/journal.pone.0027691

**Published:** 2011-11-16

**Authors:** Daniel A. Nagode, Ai-Hui Tang, Miranda A. Karson, Matthias Klugmann, Bradley E. Alger

**Affiliations:** 1 Department of Physiology, University of Maryland School of Medicine, Baltimore, Maryland, United States of America; 2 Program in Molecular Medicine, Graduate Program in Life Sciences, University of Maryland, Baltimore, Maryland, United States of America; 3 Program in Neuroscience, Graduate Program in Life Sciences, University of Maryland, Baltimore, Maryland, United States of America; 4 Translational Neuroscience Facility, University of New South Wales, Sydney, New South Wales, Australia; Claremont Colleges, United States of America

## Abstract

Acetylcholine (ACh) influences a vast array of phenomena in cortical systems. It alters many ionic conductances and neuronal firing behavior, often by regulating membrane potential oscillations in populations of cells. Synaptic inhibition has crucial roles in many forms of oscillation, and cholinergic mechanisms regulate both oscillations and synaptic inhibition. *In vitro* investigations using bath-application of cholinergic receptor agonists, or bulk tissue electrical stimulation to release endogenous ACh, have led to insights into cholinergic function, but questions remain because of the relative lack of selectivity of these forms of stimulation. To investigate the effects of selective release of ACh on interneurons and oscillations, we used an optogenetic approach in which the light-sensitive non-selective cation channel, Channelrhodopsin2 (ChR2), was virally delivered to cholinergic projection neurons in the medial septum/diagonal band of Broca (MS/DBB) of adult mice expressing Cre-recombinase under the control of the choline-acetyltransferase (ChAT) promoter. Acute hippocampal slices obtained from these animals weeks later revealed ChR2 expression in cholinergic axons. Brief trains of blue light pulses delivered to untreated slices initiated bursts of ACh-evoked, inhibitory post-synaptic currents (L-IPSCs) in CA1 pyramidal cells that lasted for 10's of seconds after the light stimulation ceased. L-IPSC occurred more reliably in slices treated with eserine and a very low concentration of 4-AP, which were therefore used in most experiments. The rhythmic, L-IPSCs were driven primarily by muscarinic ACh receptors (mAChRs), and could be suppressed by endocannabinoid release from pyramidal cells. Finally, low-frequency oscillations (LFOs) of local field potentials (LFPs) were significantly cross-correlated with the L-IPSCs, and reversal of the LFPs near *s. pyramidale* confirmed that the LFPs were driven by perisomatic inhibition. This optogenetic approach may be a useful complementary technique in future investigations of endogenous ACh effects.

## Introduction

Pyramidal cell firing in cortical systems, including hippocampus, is directly controlled by perisomatic inhibition [1] mediated primarily by parvalbumin (PV)- or cholecystokinin (CCK)-expressing interneurons [2]. Bath-application of high (≥10 µM) concentrations of cholinergic agonists such as carbachol (CCh) induces oscillations with strong inhibitory synaptic components and is often used in *in vitro* studies [3], [4], [5], [6]. However, bath-application cannot mimic the temporal or spatial characteristics of *in vivo* ACh release, which acts relatively rapidly in restricted regions with decreasing concentrations as it travels from its axonal release sites to non-synaptic receptors via ‘volume conduction’ [7], [8]. Bulk extracellular electrical stimulation in acute slices releases ACh that activates interneurons [9], [10], [11], yet bulk stimulation will also affect non-cholinergic fibers and glia, and it is not clear whether the same effects can be produced by endogenous ACh. The IPSC oscillations induced by bath-application are predominantly caused by muscarinic ACh receptor (mAChR) activation, and it is not known if axonally released ACh can do the same.

Perisomatic inhibition has well-established roles in gamma and in certain theta rhythm oscillations [12], [13], including those which are sensitive to the mAChR antagonist, atropine (“atropine- sensitive” theta). Theta is a basic operational mode of the hippocampus [12], although its underlying mechanisms are not fully understood. While true theta cannot be duplicated *in vitro*, model forms of oscillation, designated “low-frequency oscillations” (LFOs), can be investigated in slices. Perisomatic inhibition in the hippocampus is mediated either by CCK-expressing interneurons, which express the type 1 cannabinoid receptors (CB1Rs) [14], [15], [16] or PV-expressing interneurons, which do not. PV cells are well known to generate hippocampal rhythms, whereas CCK cells are thought to be less directly involved and to serve in a modulatory capacity [2]. However, IPSP/Cs induced in CA1 pyramidal cells by global mAChR activation are generally sensitive to suppression by the retrograde signaling process called depolarization-induced suppression of inhibition (DSI) [17], [18], [19], [20], which is mediated by endocannabinoids [15], [21], [22], [23]. The DSI-sensitive IPSP/C activity induced by bath-applied mAChR agonists is often oscillatory at 4–7 Hz [19], [20], [24]. Therefore, the CB1R+ (CCK) cells are probably a major source of the rhythmic IPSCs generated by bath-applied mAChR agonists. The question remains whether axonally released ACh is capable of similarly activating rhythmic IPSCs from CB1R+ cells, or whether their participation in previous work was an artifact of the bath-application technique. Demonstration of ACh-induced IPSCs arising from CB1R+ cells would not establish that the cells generate rhythmic IPSCs *in vivo*, but would show that such a function is at least possible.

Fundamental questions remaining unanswered therefore include: 1) whether the large-amplitude, endocannabinoid-sensitive, rhythmic IPSC activity that occurs with abrupt onset in CCh-treated slices is also triggered by axonally-released ACh, 2) whether these IPSCs can serve as “current generators” for local field potential oscillations [12], [13] in CA1, and 3) whether selective stimulation of ACh release can rapidly initiate inhibitory LFOs independently of activation of pyramidal cells or glutamatergic synapses.

Optogenetic methods [25] can help address these questions. Therefore, we genetically targeted ChR2 to cholinergic projection cells of the mouse MS/DBB with viral vectors and Cre-Lox technology [26], [27]. Using blue-light stimulation to release ACh from cholinergic fibers in acute hippocampal slices from these animals, we triggered bursts of IPSCs in the CA1 region, with ionotropic GluRs (iGluRs) blocked. Brief trains of light flashes induced bouts of rhythmic, endocannabinoid-sensitive IPSCs lasting 10's of seconds, accompanied by LFOs that were detected as local field potentials (LFPs). IPSCs and LFPs were abolished by GABA_A_R or mAChR antagonists, and were synchronous across wide areas of *s. pyramidale*. LFPs were well correlated with IPSCs, and reversed polarity near *s. pyramidale*. These results indicate that selective release of ACh in the hippocampus can generate rhythmic IPSCs and LFOs by driving perisomatic-targeting, CB1R+ interneurons.

## Materials and Methods

### Transgenic mice and virus injection

**Figure 1:**
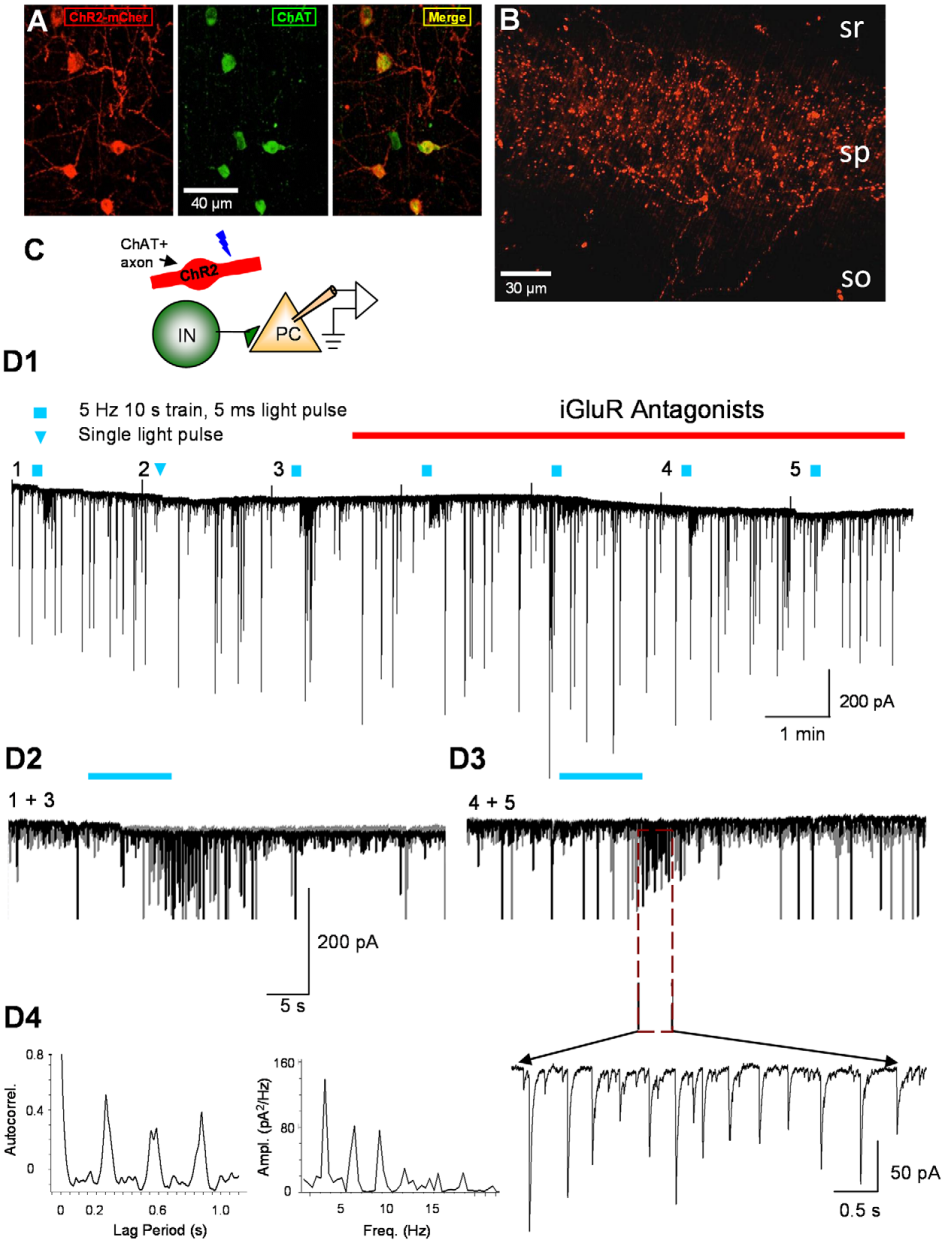
ChR2- mCherry expression in cholinergic MS/DBB projection neurons and light-induced IPSCs in CA1 pyramidal cells. A) Tissue section through the MS/DBB showing ChR2-mCherry, immunostaining for ChAT, and the merged image. B) Section of the hippocampus showing ChR2-mCherry expression in axons. sr  =  *s. radiatum*; sp  =  *s. pyramidale*; so  =  *s. oriens*. C) Schematic drawing of the recording arrangement. D1) Continuous recording from a CA1 pyramidal cell in a ChR2-expressing slice. Repeated 10-s trains of 5-ms blue light flashes repeated at 2-min intervals (squares) elicited bursts of L-IPSCs (downward deflections). A single light pulse (downward triangle) had no obvious effect. The small upward deflections (truncated in the illustration) are capacitive transients produced by conductance pulses given to the cell. D2) Two trials (1 and 3 in D1) in the absence of iGluR antagonists were aligned at the time of the light train and overlapped. D3) Two trials (4 and 5 in D1) in the presence of iGluR antagonists, 5 µM NBQX plus 5 µM CGP37849, were aligned and overlapped. An expanded portion of the trace (below) reveals the occurrence of rhythmic L-IPSCs after the end of the light train. (The traces in D2 and D3 are shown at a larger amplitude scale than in D1, and the largest IPSCs are cut off.) The autocorrelation function (D4, left) and power spectrum (D4, right) illustrate the regularity (peak frequency ∼3 Hz) of L-IPSC activity from this cell.

All animal handling procedures were in accordance with national and international guidelines and were approved by the University of Maryland School of Medicine IACUC (Approval #0609001). Mice expressing Cre recombinase under the control of the ChAT promoter [ChAT-Cre mice (B6;129S6-Chat^tm1(cre)Lowl^/J)] were obtained from The Jackson Laboratory. Homozygous mice were bred and maintained at UMSOM. The plasmid pAAV-EF1a-double floxed-hChR2 (H134R)-mCherry-WPRE-HGHpA (Addgene stock # 20297) was packaged into a pseudotype adeno-associated virus (AAV) 2/1 chimeric vector [28] (“AAV-ChR2-mCherry”) for *in vivo* delivery into the mice. A Cre-dependent “FLEX” switch [29] ensures that ChR2 expression occurs only in cholinergic cells [30] (Fig. 1A). In a few experiments, AAV vectors of serotype 2/1, 2/5, or 2/9 containing the same plasmid were used (University of Pennsylvania Vector Core, Lot # V1447, V1449, and V1534, respectively). All vectors had a titer of >10^12^ genome copies/ml. Mice, ≥8 wks old, were sedated, anesthetized, and stereotactically infused with 1–1.5 µl of AAV-ChR2-mCherry into the MS/DBB at 0.1 µl/min with a glass micropipette. Hippocampal slices were prepared from ChAT-Cre mice ≥5 weeks later.

### Slice Preparation

Mice were sedated with isoflurane and decapitated. Transverse hippocampal slices (400 µm thick) were cut in ice-cold artificial cerebrospinal fluid (ACSF) on a Vibratome VT1200S (Leica). Slices recovered in a humidified interface holding chamber at room temperature (∼22°C) for ≥1.5 h before use. ACSF contained (mM): 120 NaCl, 3 KCl, 2 MgSO_4_, 2.5 CaCl_2_, 1 NaH_2_PO_4_, 25 NaHCO_3_, 20 glucose, and was bubbled with 95% O_2_ and 5% CO_2_.

### Optogenetic Experiments

For ChR2 excitation, square pulses of blue light (450–490 nm, 5 ms in duration) were delivered through the 60X or 40X water-immersion objective of a Nikon E600 microscope to the entire visual field centered on the recorded cell. A 100 W mercury lamp (Nikon C-SHG1) or 300 W Xenon lamp (PerkinElmer) served as a light source. Measured light power at the microscope objectives was 300-500 µW. To set light-pulse and train duration, a high-speed shutter (Uniblitz VMM-D1, Vincent Associates) or a Lambda DG4 wavelength switcher (Sutter Instruments) was controlled by a Pulsemaster A300 digital timer (World Precision Instruments), which was triggered by pClamp10 software (Molecular Devices). mCherry was visualized under 557–597 nm light.

### Electrophysiology

Slices were transferred to a submersion chamber (model RC-27, Warner Instruments) and continuously perfused with ACSF at 22°C. Voltage-clamped IPSCs were recorded at a holding potential of -70 mV with patch pipettes filled with (mM) 85 Cs-methanesulfonate, 50 CsCl, 10 Na-HEPES, 3 ATP-Mg, 0.3 GTP-Tris, 0.1 CaCl_2_, 1 Cs_4_-BAPTA, 5 QX-314, and 1 MgCl_2_ (pH 7.2, 290–300 mOsm). Pipettes had resistances of 3–6 MΩ in the bath and access resistance was continually checked; if it changed by >20%, data were discarded. LFPs in CA1 were recorded with pipettes filled with normal ACSF. Axopatch 200B amplifiers (Molecular Devices) were used; signals were filtered at 2 kHz and digitized at 5–10 kHz with a Digidata 1440A interface and Clampex 10 software. LFPs were filtered between 1–20 Hz. Chemicals were purchased from Sigma (St. Louis, MO) except for NBQX (Ascent Scientific), CGP37849 and mecamylamine hydrocholride (Tocris Biosciences). Goat anti-ChAT antibody (AB144P) was purchased from Millipore Bioscience Research Reagents and used at a concentration of 1:1000.

### Statistical Analysis

Data were analyzed with Clampfit10 and Mini-analysis (Synaptosoft Inc.). DSI of L-IPSCs was calculated as the ratio of integrated current within a 5-s window [16] ∼10-s after a depolarization step (to avoid the large inward current artifact that would otherwise interfere with the charge calculation), compared to the same quantity taken immediately before the step, normalized for the transience of the L-IPSC bursts; ≥2 DSI trials were averaged per cell. Data are given as mean ± SEM. Significance levels for paired t-tests are indicated.

## Results

**Figure 2:**
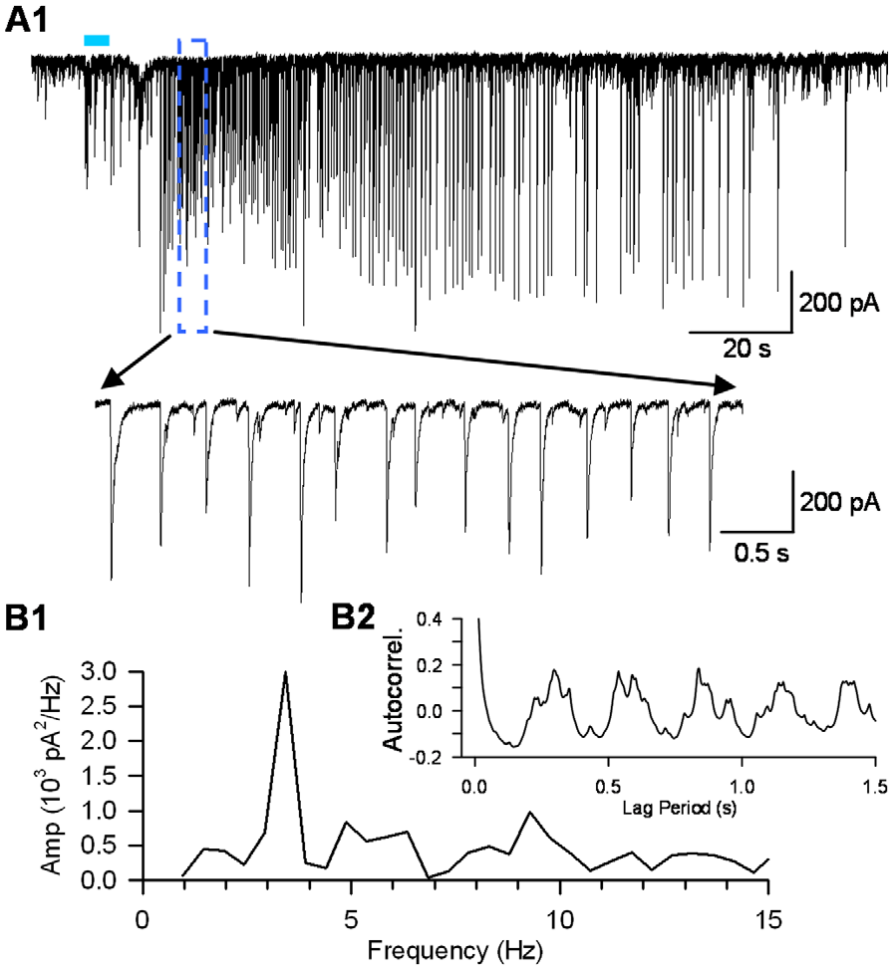
L- IPSCs oscillate rhythmically at low frequency in eserine and 4-AP. Results from a typical pyramidal cell recorded in the presence of the cholinesterase inhibitor, eserine, 1 µM, and a low concentration of the voltage-gated K^+^ channel blocker 4-AP, 20 µM, in the extracellular solution. A train (5-ms light pulses, 5 Hz, 5 s) was delivered during the horizontal blue line. A) After a delay of several seconds a burst of large L-IPSCs began and persisted for >1 min. The IPSCs occurred rhythmically, with a peak frequency of ∼3 Hz (power spectrum in B1, autocorrelogram in B2). Eserine and 4-AP were present in all subsequent experiments.

To test the hypothesis that selective release of ACh activates hippocampal interneurons, we recorded from CA1 pyramidal cells in hippocampal slices having ChR2-mCherry labeled cholinergic axons (Fig. 1B) and delivered trains of blue light pulses (1–10 s/5 Hz, 5-ms pulse duration). In the presence of iGluR antagonists, light trains could induce bursts of large, rhythmic IPSCs that greatly outlasted the light stimulation (e.g., Fig. 1D). These are considered light-evoked, or L-IPSCs, even though their occurrence did not depend on continuous light stimulation. We observed L-IPSCs in 9 cells, of which data from 7 were sufficient to permit analysis. The L-IPSC bursts occurred near or slightly after the end of the light stimulation (onset latency 9.4±3.48 s, n = 7 cells). The baseline IPSC frequency of 4.5±1.33 Hz and amplitude, 19.1±1.70 pA, increased to 5.3±1.34 Hz and 52.8±16.98 pA, respectively, during the bursts. The duration of the L-IPSC bursts varied between 11.0 and 65.5 s (33.7±9.38 s). Hence, light stimulation of cholinergic axons causes activation of CA1 interneurons. Unfortunately, there was significant trial-to-trial and cell-to-cell variability, and success rate in obtaining such records was unsatisfactory (we observed clear L-IPSC responses in only ∼10% of the cells recorded from). The low success rate is presumably related to the variable truncation of MS/DBB axons that were superficial enough to be activated by light. Although there was no obvious, strong relationship between mCherry fluorescence intensity and success in eliciting ACh responses, we do not have detailed information on the proximity of the ACh release sites and the cholinergic receptors on the interneurons, which must be a major variable. These results demonstrate the basic concepts that optogenetically released ACh activates CA1 interneurons and induces rhythmic L-IPSCs that are independent of ionotropic glutamate receptors. However, the variability in the results precluded an efficient investigation. To enhance the success rate, we applied the cholinesterase inhibitor, eserine (1 µM), to permit the ACh to diffuse farther from its release sites, and a low (10-20 µM) concentration of the K^+^-channel antagonist, 4-AP, to enhance the ChR2-induced neurotransmitter release [31], [32]. Under these conditions robust, repeatable L-IPSC bursts were evoked in 22/25 cells (88%, e.g. Fig. 2A). Apart from increasing the success rate and slightly affecting the responses quantitatively, the presence of eserine and 4-AP did not appear to affect the IPSC oscillations qualitatively; the large IPSC amplitudes and oscillation frequencies with the drugs present were similar to the responses evoked in their absence (see below). It is likely that the drugs alter the system quantitatively and therefore preclude a simple translation of these results to the *in vivo* case, however. Comparison of data from an untreated slice (Fig. 1) and a treated slice (Fig. 2) showed peak spectral power in the range of ∼3 Hz (Figs. 1D4, 2B1), which was similar to the peaks observed in the autocorrelograms (Figs. 1D4, 2B2). Eserine and a low concentration of 4-AP were present in all subsequent experiments.

**Figure 3:**
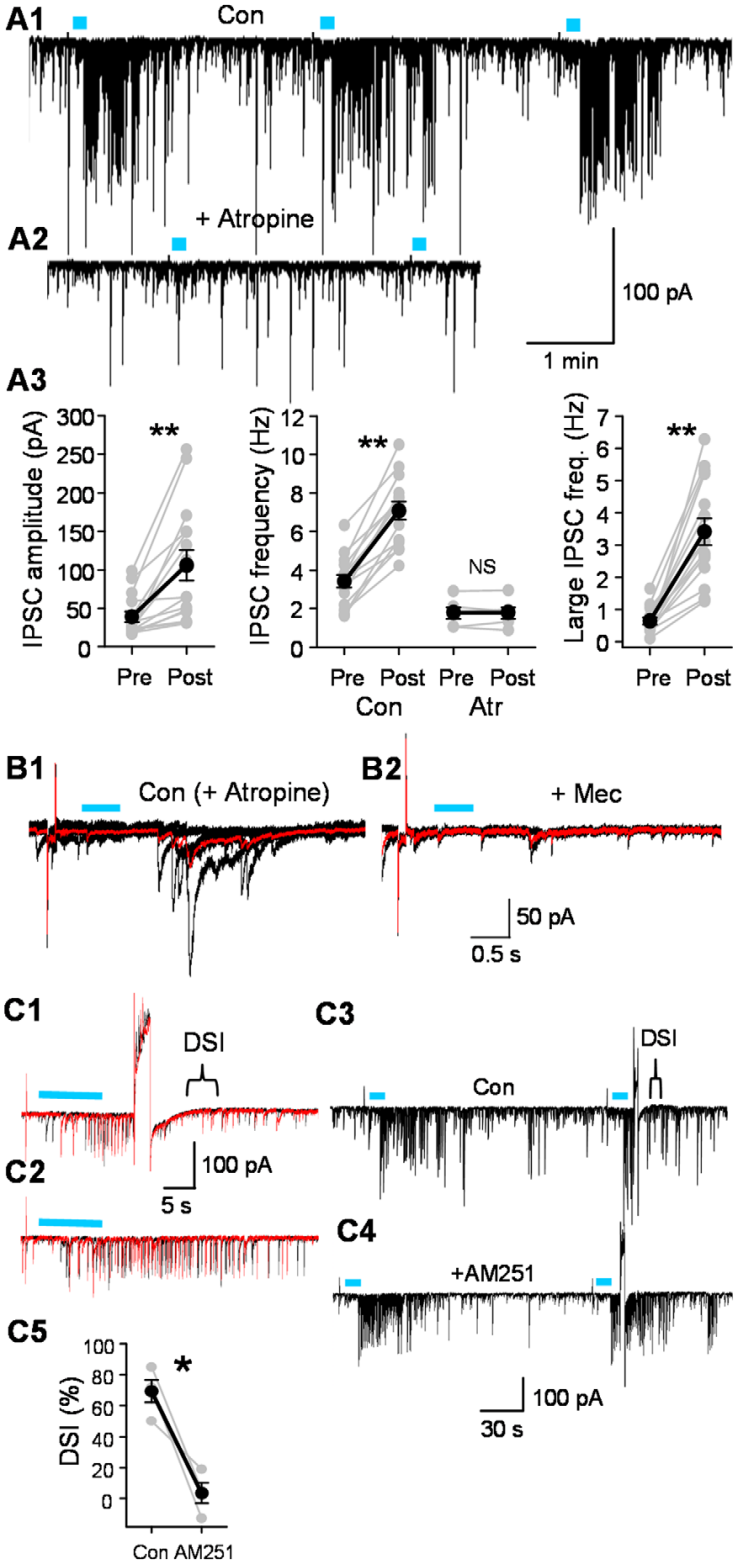
L- IPSCs are triggered primarily by mAChRs and reduced by endocannabinoids. A) Repeated light trains (5 Hz, 10 s, 5-ms pulses) elicited IPSC bursts (A1) that were nearly abolished by atropine (A2). A3) Group data of IPSCs in basal (Pre) conditions, and after light stimulation (Post). Values of amplitudes and frequency for all IPSCs (left and middle graphs), and frequency of large IPSCs (i.e., two s.d.s > the mean basal IPSC; right graph). B1) In atropine, light trains (10 Hz, 0.5 s) elicited small IPSC bursts (5 traces, mean in red) that were reduced by the non-selective nAChR antagonist, mecamylamine, 10 µM (B2, 2 traces, different cell than A1, A2). C1) L-IPSCs recorded in a CA1 pyramidal cell elicited by a 5 Hz, 10-s train of light pulses. DSI was induced by a 3-s voltage step (to +20 mV). C2) Control trials show IPSC bursts in the absence of DSI. C3, C4) DSI of L-IPSCs was blocked by the CB1R antagonist, AM251, 5 µM (different cell than C1, C2). C5) Group data (n = 4) of DSI in control conditions and after bath application of AM251; ^*^  =  p<0.05; ^**^  =  p<0.01.

Large L-IPSC bursts with similar time courses could be repeatedly evoked in a given cell (e.g. Fig. 3A1), had onset latencies of 9.5±2.21 s from the beginning of the light train and lasted for 72.5±7.90 s (n = 11 cells). The L-IPSC amplitude was 105.5±19.59 pA (vs. basal IPSCs, 38.8±6.98 pA, n = 15 cells, p<0.01, Fig. 3A3, left graph). The larger size of the IPSCs and longer duration of the responses apparently reflected the effects of eserine and 4-AP. As noted, however, the overall effects of the light stimulation were qualitatively similar to the untreated condition. The frequency of all IPSCs was increased by light stimulation from 3.4±0.33 Hz (basal) to 7.1±0.46 Hz during the light-induced bursts (n = 15, p<0.01, Fig. 3A3, middle graph). The increase in large L-IPSCs appeared especially dramatic. Large hippocampal IPSCs often represent the summation of smaller IPSCs (e.g., [33]) and this is especially true of IPSCs originating from CCK-interneurons [34]. Increases in large IPSCs may represent enhanced synchrony of release from these interneurons. We cannot distinguish the small IPSCs that may act as subcomponents of the large ones from other small IPSCs in these experiments, however. Reanalysis of the data with a threshold of 1.96X the mean basal IPSC (to capture the largest IPSCs, those at least 2 s.d. greater than the mean) revealed that the frequency of large L-IPSCs increased from 0.6±0.11 Hz (basal) to 3.4±0.41 Hz after light stimulation (n = 15, p<0.01, Fig. 3A3, right graph). The general mAChR antagonist, atropine, 2 µM, prevented the occurrence of most L-IPSCs (Figs. 3A2, 3A3, middle graph, n = 6 cells). As we have recently reported [35], in atropine brief bursts of small L-IPSCs could be recorded in 9 of 16 cells (e.g., Fig. 3B1), and these were virtually abolished by the non-selective nAChR antagonist, mecamylamine (Fig. 3B2). We did not investigate nAChR-dependent events further in this study. The GABA_A_R antagonist, gabazine, blocked all activity (n = 3 cells, not shown), confirming that the responses are entirely IPSCs (iGluR antagonists were present). Thus light-elicited ACh release can induce IPSCs in pyramidal cells via a relatively prolonged mAChR action and perhaps in some cases a shorter nAChR effect. We focused on the mAChR-induced L-IPSCs.

To test the hypothesis that L-IPSCs mainly arise from CB1R+ interneurons under our conditions, we evaluated their susceptibility to DSI. Voltage steps (from the holding potential of −70 mV to a value from 0 to +30 mV, lasting 2 or 3 s, depending on the cell) were used to elicit DSI. L-IPSC DSI was evaluated by comparing the current trace integrals (a measure that includes all L-IPSCs and not just the largest ones) before and after the voltage step (see Methods). We observed that L-IPSCs were suppressed by 69.5±7.19% (p<0.05, n = 10, Fig. 3C). The CB1R antagonist, AM251, prevented the suppression (3.5±6.54%, n = 4, n.s.), confirming that it was endocannabinoid-dependent. Hence, the majority of L-IPSCs originate in CB1R+ interneurons.

**Figure 4:**
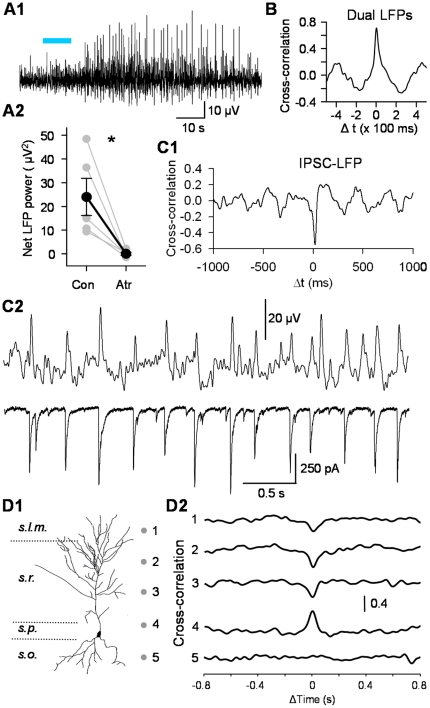
ACh-release-induced rhythmic LFPs are well correlated with L- IPSCs. A1) Extracellular recording in *s. pyramidale.* Light trains (5 Hz, 10 s) elicited bursts of LFPs. A2) Group data showing increase in total LFP power obtained by integrating the spectral analysis over the range of 2–12 Hz, before and after atropine application (n = 5, *p<0.05). B) As in (A) except that two extracellular electrodes were placed ∼200 µm apart. The plot shows that the two L-LFPs were temporally cross-correlated. C) Simultaneous recordings of L-IPSCs and LFPs recorded ∼200 µm away. C1) Cross-correlation plot indicates L-IPSC peak occurs very near the LFP peak. C2) Sample traces of simultaneous L-IPSC, L-LFP recordings. D) Sink-source analysis of L-LFPs. D1) Dots indicate recording locations. D2) Two extracellular electrodes recorded L-LFPs at locations 1 – 5; traces show LFP cross-correlations versus the simultaneous LFP recorded in *s. pyramidale* (4). The records suggest an LFP current source near 4.

Direct electrical stimulation of perisomatic-targeting interneurons can synchronize pyramidal cell firing [36]. Perisomatic-targeting interneurons have wide-ranging axonal projections, with a single cell synapsing on hundreds of pyramidal cells (e.g., [37]). If the L-IPSCs originate from perisomatic-targeting interneurons, they should affect the CA1 cell population over a wide area even with iGluR antagonists present. To test this hypothesis, we recorded LFPs with extracellular electrodes and delivered light stimulation. Light trains induced rhythmic bursts of LFPs (L-LFPs) in 16/22 (73%) of experiments (Fig. 4A1). The rhythmic L-LFPs were abolished by gabazine, confirming that they were driven by GABA_A_R responses (n = 3, not shown). Spectral analysis of the LFPs shows that the bursts of rhythmic activity occurred at a peak frequency of ∼3 Hz. Following light stimulation, total power in the range of 2–12 Hz was increased by 28.9±6.69 µV^2^ (n = 9) and this increase was essentially abolished by atropine (0.03±0.63 µV^2^ over baseline power, n = 5, Fig. 4A2).

LFPs occurred across a broad area of the CA1 region because recordings with two field potential electrodes separated by ∼200 µm detected a strong cross-correlation (e.g., Fig. 4B). If the L-LFPs are indicative of the extracellular fields generated by perisomatic targeting interneurons, then the rhythmic L-LFPs and L-IPSCs should also be closely related. This prediction was supported by cross-correlation analysis of simultaneously recorded L-LFPs and L-IPSCs (Fig. 4C); similar results obtained in 9/10 slices. A final prediction of the hypothesis that perisomatic interneurons drive the L-LFPs is that a current source density analysis should show a reversal in or near *s. pyramidale*. Synchronous inhibitory inputs to a population of pyramidal cell somata creates a current source in *s. pyramidale*, i.e., outward currents flowing during the perisomatic IPSPs which are detected as local positive waves, while the dendritic regions serve as current sinks [12], [13]. Indeed, simultaneous recordings with two field electrodes, one in *s. pyramidale* and one moved to various positions in *s. radiatum* or *s. oriens*, confirmed this prediction. The field oscillations were highly correlated, but ∼ 180° out of phase (Fig. 4D), with positive peaks in *s. pyramidale* occurring simultaneously with negative peaks in the dendritic regions; similar results obtained in 3/3 slices tested.

## Discussion

We used an optogenetic approach to test the hypothesis that ACh released from cholinergic axons in CA1 can activate perisomatic-targeting interneurons, including CB1R+ cells, and that the resulting IPSPs affect CA1 field potential oscillations. The results are consistent with the hypothesis. mAChR-induced IPSC activity was driven independently of iGluRs [3], septal GABAergic afferents [38] or modulators that might be released by electrical stimulation. We cannot rule out that ACh itself may have influenced the release of additional modulators that in turn affected the interneurons, but this concern will apply to essentially all past investigations of ACh action as well. Further investigation would be required to address the issue conclusively. LFOs induced by ACh release were detectable both in whole-cell IPSCs and extracellularly recorded LFPs, and ACh mainly affected CB1R+ interneurons via relatively long-lasting mAChR-mediated effects, although brief bursts of nAChR-mediated L-IPSCs, and occasional large DSI-insensitive IPSCs also occurred. Hence the occurrence of DSI-sensitive oscillatory IPSCs was not an artifact of bath-application methods in earlier work (e.g., [20], [24]. nAChRs can influence the patterning of network activity *in vitro* [39] and GABA release [27], [35] and it will be important to elucidate the role of nAChRs in LFOs triggered by endogenous ACh in future work.

Despite the relatively low success rate in obtaining robust ACh-induced IPSC activity in eserine- and 4-AP-untreated slices, the facts that: a) such activity could be induced and, b) the properties of the responses that occurred in untreated slices were qualitatively similar to those in treated slices, demonstrate that the responses were not created by the drugs, but represent a fundamental biological capacity of the system. These results therefore show that optogenetically released ACh is sufficient to induce rhythmic bursts of inhibitory responses and LFPs. Of course, use of the slice preparation itself, and eserine and 4-AP in most experiments, means that the results cannot be assumed to translate directly to the *in vivo* case.

Even given these caveats, this model system provides some unique advantages for studying the kinetics and regulation of ACh effects on inhibition. The onset of the ACh activation of IPSCs takes place soon after beginning light stimulation, much faster than can be achieved by bath-perfusion, for example. The lag of ∼9 s before the appearance of large, rhythmic IPSCs may reflect the kinetics of the mAChR-dependent response, intrinsic interneuron biophysical properties, as well as interactions among the interneurons [40], [41], and diffusion delays across distances between the cholinergic receptors and ACh release sites.

As found with bath-application of cholinergic agonists [15], [17], [18], [42], the large L-IPSCs were sensitive to DSI (Fig. 3C), indicating that they originate primarily from CB1R+ interneurons, which in hippocampus are CCK+ [16], [43]. Selective release of ACh thus permitted independent support for the proposal [11] that CCK cells are more readily activated by endogenously released ACh than are PV cells. The PV cells are µ-opioid receptor positive (µOR+) [44] but do not express CB1R [2], [45], hence DSI sensitivity argues that the large L-IPSCs do not originate primarily from PV cells. Nevertheless, µOR-sensitive IPSCs arising from PV interneurons can be triggered by CCh application [46], and might be among the population of ACh-induced IPSCs. This possibility remains to be investigated. Ready cholinergic stimulation of CCK cells might also enable them to assist the PV cell network in initiating inhibitory oscillations [2]. CB1R- (presumed PV) cells receive numerous glutamatergic inputs and are strongly activated by glutamatergic synapses, indicating that under many conditions the PV cells primarily mediate feedforward inhibition [47]. However, our data suggest that the CCK cells could also contribute significantly to feedforward inhibition when ACh release occurs.

Besides acting as rhythm generators, interneurons are also “current generators”. IPSCs, even from a single interneuron [48], contribute significantly to LFPs [6], [12], [13]. Coupled discharges of CA2/CA3 pyramidal cells [4], and recurrent feedback loops between glutamatergic and GABAergic cells are required to generate CA3 gamma rhythms, but perisomatic inhibitory currents dominate the expression of the LFPs [6]. Interneurons in *s. oriens* driven by glutamatergic inputs participate in CA1 theta rhythm IPSCs [49] and mAChR agonists induce theta-like membrane potential oscillations in dendritic-targeting, lacunosum-moleculare interneurons [5]. Numerous other mechanisms [12], [13], [50] could contribute to driving cholinergically-mediated LFOs. Along this line, bath-application experiments [20], [24], suggested the existence of a mAChR-driven network, perhaps like the cortical network [41], that was independent of iGluRs, but that could generate rhythmic, endocannabinoid-sensitive IPSCs in pyramidal cells. The present experiments now reveal that brief and spatially restricted endogenous ACh release can engage this network, and demonstrate that it can contribute to LFO current generation in CA1. Because of its spatially restricted nature, our illumination did not activate all the interneurons that would be activated following the firing of septal afferents, and additional interneurons no doubt participate in cholinergically-generated rhythms *in vivo*. In addition, interactions between CCK-and PV-networks [51] might participate in LFO generation by ACh, but testing this possibility was well beyond the scope of the present investigation.

ACh provides a tonic, rather than a “pacemaker” input [12] and mAChR-induced oscillations might emerge from interactions within the interneuronal networks, as is the case with atropine-resistant rhythms [49], [52]. True behaviorally-relevant oscillations are highly complex, and the function of the CB1R+ network in the larger oscillatory circuitry *in vivo* remains to be elucidated.
